# Nifedipine improves blood flow and oxygen supply, but not steady-state oxygenation of tumours in perfusion pressure-controlled isolated limb perfusion

**DOI:** 10.1038/sj.bjc.6600611

**Published:** 2002-11-26

**Authors:** O Thews, M Hummel, D K Kelleher, B Lecher, P Vaupel

**Affiliations:** Institute of Physiology and Pathophysiology, University of Mainz, Duesbergweg 6, 55099 Mainz, Germany

**Keywords:** calcium channel blocker, isolated limb perfusion, nifedipine, tumour perfusion, tumour oxygenation, tumour vascular resistance

## Abstract

Isolated limb perfusion allows the direct application of therapeutic agents to a tumour-bearing extremity. The present study investigated whether the dihydropyridine-type Ca^2+^-channel blocker nifedipine could improve blood flow and oxygenation status of experimental tumours during isolated limb perfusion. Perfusion was performed by cannulation of the femoral artery and vein in rats bearing DS-sarcoma on the hind foot dorsum. Perfusion rate was adjusted to maintain a perfusion pressure of 100–140 mmHg throughout the experiment. Following equilibration, nifedipine was continuously infused for 30 min (8.3 μg min^−1^ kg^−1^ BW). During constant-pressure isolated limb perfusion, nifedipine can significantly increase perfusion rate (+100%) and RBC flux (+60%) through experimental leg tumours. ‘Steal phenomena’ in favour of the surrounding normal tissue and oedema formation were not observed. Despite the increased oxygen availability (+63%) seen upon application of this calcium channel blocker, nifedipine does not result in a substantial reduction of tumour hypoxia, most probably due to an increase in O_2_ uptake with rising O_2_ supply to the tumour-bearing hind limb. Nifedipine application during isolated limb perfusion can enhance tumour microcirculation and may therefore promote the delivery (pharmacokinetics) of anti-cancer drugs to the tumour and by this improve the efficacy of pressure-controlled isolated limb perfusion.

*British Journal of Cancer* (2002) **87**, 1462–1469. doi:10.1038/sj.bjc.6600611 www.bjcancer.com

© 2002 Cancer Research UK

## 

Isolated limb perfusion (ILP) is a treatment modality for malignancies of the extremities in which the tumour-bearing limb is isolated from the patient's circulatory system and perfused separately. This procedure allows the administration of anti-cancer agents to the tumour at high doses with only a minimum risk of systemic toxicity. High-dose regional chemotherapy was one of the first concepts in ILP which was complemented more recently by the application of cytokines and immune-modulators frequently in combination with hyperthermia (Omlor *et al*, 1995; Eggermont *et al*, 1996; Pisters *et al*, 1997; Schraffordt-Koops *et al*, 1998; Seynhaeve *et al*, 2002). Since its introduction into the clinical setting (Creech *et al*, 1958), ILP with high-dose chemotherapy alone or in combination with biological agents has been used for the treatment of loco-regionally advanced melanomas and locally advanced soft tissue sarcomas not amenable to surgical resection (for a review see Hohenberger and Kettelhack, 1998).

The response to chemo- or cytokine-therapy may however be compromised by ‘biological’ factors such as tumour blood flow and microcirculation, tissue oxygenation or pH distribution. The individual tumour microenvironment is a paramount determinant in the outcome of non-surgical treatments (Vaupel *et al*, 1989; Höckel and Vaupel, 2001). For example, bulky tumours, which are an indication for ILP in sarcoma treatment, often show a progressive deterioration and heterogeneity of tumour blood flow during growth which in turn is responsible for diffusion- and perfusion-limited hypoxia resulting in a reduced efficacy of oxygen-dependent agents (e.g., anthracyclines, cyclophosphamide or melphalan; Vaupel *et al*, 2001). At the same time a poor and inhomogeneous delivery of anti-cancer agents will diminish the cytotoxic effect. For this reason, supportive treatment modalities might be helpful which lead to an improvement or homogenisation of tumour blood flow and/or a reduction of hypoxia already present in many experimental or human tumours.

One group of drugs that might achieve these goals (and by this might increase the efficacy of ILP) are calcium channel blockers (CCB). In experimental tumour therapy, CCBs have been of broad interest because of their ability to modify tumour blood flow and improve oxygenation status.

Blood flow modification following administration of these agents results from a dilation of resistance vessels and a reduction in blood viscosity. Over the last two decades, CCBs (e.g., flunarizine) have been shown to improve tumour blood flow, oxygen availability (Kaelin *et al*, 1984; Vaupel and Menke, 1987, 1989), tumour oxygenation (Dewhirst *et al*, 1992) and to increase tumour radiosensitivity (Hill and Stirling, 1987; Wood and Hirst, 1988, 1989). However, the proposed increase in tumour blood flow resulting from a vasodilatation of vessels feeding the tumour is strongly dependent on the maintenance of a stable perfusion pressure which could not be achieved with all types of CCBs. This might however not be a problem in the case of the isolated perfusion of tumour-bearing limbs by an extracorporeal circuit as performed clinically, since perfusion pressure can be regulated by enhancing or lowering perfusate flow rate by means of an external pump. Thus, a CCB-induced drop in perfusion pressure could easily be compensated by an increase in the perfusion rate. Besides their impact on the tumour oxygenation via an increase in the O_2_ supply, CCBs also seem to be able to improve the O_2_ status directly by reducing oxygen consumption (Biaglow *et al*, 1986; Vaupel and Mueller-Klieser, 1986).

In addition, CCBs show a chemosensitising effect (Helson, 1984) which is thought to be independent of effects on the classical slow inward calcium channel. The ability of verapamil and other CCBs to reverse multi-drug resistance is linked to the interaction of the CCBs with the P-glycoprotein in the membrane of resistant tumour cells (Cornwell *et al*, 1987). The enhanced anti-tumour and anti-metastatic potential of cisplatin in combination with nifedipine in mice was attributed to the inhibition of tumour cell-platelet aggregation and inhibition of platelet-enhanced tumour cell adhesion to endothelial cells *in vitro* and *in vivo* by CCBs of the dihydropyridine type (Honn *et al*, 1985; Onoda *et al*, 1989).

The present study investigated the possibility of using nifedipine to improve perfusion and oxygenation status of experimental tumours during pressure-controlled ILP and thus to possibly enhance the efficacy of chemotherapy. Nifedipine was chosen rather than other CCBs since dihydropyridine derivatives preferentially block calcium channels in the plasma membrane of arterial smooth muscle cells rather than the myocardium and therefore function in contrast to other classes of CCBs predominantly as vasodilators (Jirtle, 1988; Robertson and Robertson, 1996), a property which may be particularly suitable for increasing perfusion rate in a pressure-controlled system.

## MATERIALS AND METHODS

### Animals and tumours

Male Sprague Dawley rats (Charles River Deutschland, Sulzfeld, Germany; body weight 200–320 g) housed in our animal care facility were used in this study. They received a standard diet and acidified water *ad libitum*. Experimental tumours were grown following subcutaneous injection of DS-ascites cells (0.4 ml; approximately 10^4^ cells per μl) into the dorsum of the hind foot. Tumours were used when they reached a volume of between 0.8 and 1.3 ml, 4 to 9 days after tumour implantation corresponding to a tumour mass of 0.5% of the body weight (Workman *et al*, 1998). Tumours were implanted on both feet whereby one leg was used for isolated limb perfusion and the contralateral leg as an additional control for the metabolic and bioenergetic parameters. Studies had previously been approved by the regional ethics committee and were conducted according to UKCCCR guidelines (Workman *et al*, 1998) and to the German Law for Animal Protection.

### Nifedipine

Under protection from direct light, 2 mg nifedipine (Sigma, Deisenhofen, Germany) was dissolved in 10 ml 96% ethanol. Further dilution was made with isotonic saline resulting in a concentration of 0.1 mg ml^−1^. This stock solution was added continuously at a rate of 0.5 ml h^−1^ kg^−1^ body weight (Harvard infusion/withdrawal pump, Harvard, Edenbridge, UK) to the perfusion medium via a catheter placed in the femoral artery resulting in a nifedipine dose of 8.3 μg nifedipine min^−1^kg^−1^ body weight. An equivalent volume of the vehicle was infused into control animals.

### Isolated limb perfusion

**Figure 1:**
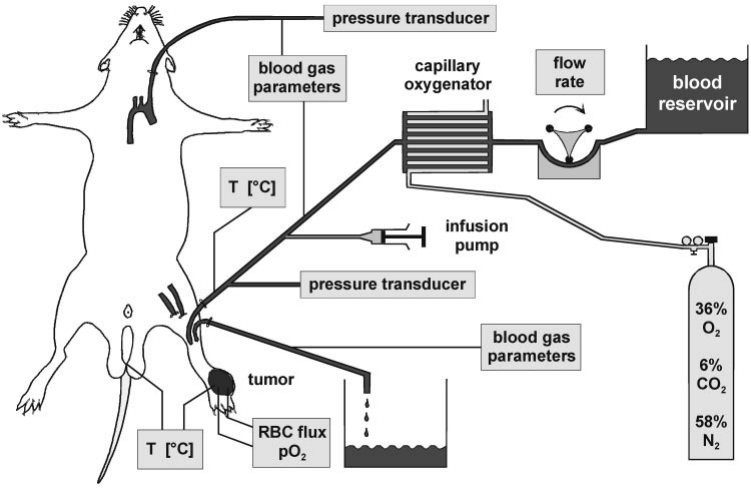
Experimental set-up for isolated limb perfusion in the rat.

A *single-pass ILP* of the tumour-bearing extremity was performed (in contrast to the earlier attempts of Nagel *et al* (1987) where a closed blood circuit was used) using a perfusion system consisting of a peristaltic roller pump (mp13GJ-4, Ismatec, Zürich, Switzerland), a capillary oxygenator (SPS40002-P, Fresenius, Bad Homburg, Germany), a water-filled heat exchanger used to maintain a perfusate blood temperature of 36.5 to 37.5°C, polyethylene cannulas and silicone tubing (Figure 1

). The perfusion medium was heparinised blood (20 i.u. ml^−1^ whole blood) obtained from donor rats. Prior to use, blood was diluted to a haematocrit of 25% with bicarbonate-buffered oxypolygelatine (55 g l^−1^, Gelifundol, Biotest Pharma, Dreieich, Germany), an isotonic colloidal blood plasma substitute solution which is used in the clinical setting. By using this drug for haemodilution an isotonic and isooncotic perfusate was obtained. Mean haemoglobin concentration (cHb) was 77 g l^−1^. Mean glucose and lactate levels were 6.1 and 6.9 mM, respectively. Blood was oxygenated with a humidified gas mixture containing 6% CO_2_, 36% O_2_ and 58% N_2_ (v v^−1^).

When tumours had reached the target volume, animals underwent general anaesthesia (sodium pentobarbital, 40 mg kg^−1^ i.p., Narcoren™, Merial, Hallbergmoos, Germany) and polyethylene catheters were surgically placed into the thoracic aorta via the left common carotid artery and into the right external jugular vein. Mean arterial blood pressure (MABP) was continually monitored through the connection of the arterial catheter to a Statham pressure transducer (type P23 ID, Gould, Oxnard, CA, USA). Arterial blood gas analysis was performed before and during ILP experiments using a pH/blood gas analyser (type ABL 5, Radiometer, Copenhagen, Denmark). Since the anaesthetic agent used is known to induce respiratory depression in high concentrations, the depth of anaesthesia was assessed by monitoring arterial blood pressure and blood gas status to ensure that these parameters remained within the physiological range throughout the experimental period.

Heparin (100 i.u. kg^−1^, body weight) was injected intravenously to prevent coagulation. An incision was made in one groin and the femoral artery and vein were exposed. After ligation of accessible collaterals, the femoral vessels were cannulated (Vasofix braunule 20 G, B Braun Melsungen, Melsungen, Germany, and Abbocath-T, 20 G, Abbott Ireland, Sligo, Ireland, respectively), flushed with 2 ml of warmed oxypolygelatine and then connected to the perfusion equipment. Blood flow in deep-seated collaterals was restricted by a groin tourniquet which was tightened upon commencement of the perfusion. Perfusate flow rate was varied (range: 0.27–2.63 ml min^−1^) to achieve a constant perfusion pressure (PP) of approximately 100 to 140 mmHg in order to maintain an adequate tissue perfusion which might otherwise be impaired with PP approximately 15 mmHg below the systemic MABP (Fontijne *et al*, 1985a,b). Perfusion pressure in the isolated leg was continuously monitored through the connection of the femoral artery to a Statham pressure transducer via a three-way stopcock.

The duration of isolated perfusion of approximately 60 min including 30 min of drug application was chosen in accordance with clinical studies and with results dealing with optimum conditions for ILP in the animal model (de Wilt *et al*, 1999). Throughout all experiments, animals lay supine on a heated operating pad and rectal temperature was maintained at 37.5–38.5°C. Animals breathed room air spontaneously.

### Laser doppler flowmetry

A multi-channel laser Doppler perfusion monitor (semiconductor laser diode, wavelength 780 nm, output power 1–2.5 mW, cut-off frequency 15 Hz, Oxford Array, Oxford Optronix, Oxford, UK) was used to measure red blood cell flux (RBC flux). Details of this method have been described earlier by Kelleher *et al* (1995, 1998b). This method uses the Doppler shift (i.e. the frequency change that light undergoes when reflected by objects in motion, such as RBCs) and has been proposed to be a valid method for the monitoring of microcirculatory function in small, discrete tissue areas (for a review see Smits *et al*, 1986). The measured flux predominantly represents the RBC flux within the illuminated volume, regardless of flow direction, and is defined as the product of the local velocity and concentration of RBCs in the measured volume which encompasses a hemisphere with a radius of approximately 0.1 mm. RBC flux signals were obtained from up to two peripheral and one central locations within the tumour using needle probes (Model array NP, o.d. 0.4 mm). A small skin incision was made with a 24-gauge needle for insertion of the needle probe so that bleeding from the wound was minimised. Total backscattered light was also recorded during the monitoring period to optimise probe positioning, minimise tissue compression (which might impair circulatory function) and ensure a constant probe location. Flux artefacts, due to alteration of the probe position (e.g., as a result of movement), additionally result in sudden changes of the total backscattered light. In the few instances where this occurred, the flux values concerned were excluded from the final evaluation. At the end of the experiment, the laser Doppler probes were left in place, the animal given an overdose of anaesthetic, the cannula in the femoral artery disconnected from the perfusion equipment and the ‘biological zero’ laser Doppler signal was established and subtracted from flux values which were then expressed as relative RBC flux and represent percentage values related to the RBC flux value determined immediately prior to nifedipine application.

Although, attempts were made to maintain the PP at a constant level during ILP, slight pressure changes (±5–10 mmHg) occurred. In order to assess whether changes in RBC flux were due to variations in PP or the result of nifedipine-induced vasodilation, the relative tumour vascular resistance (TVR) was calculated as a measure of the resistance to flow. The TVR was defined by the ratio of the MABP (or PP) and the RBC flux. This parameter is suitable for assessment of changes in the vascular diameter from variations in tumour blood flow.

### Tumour oxygen tension

Mean tumour oxygen partial pressure (pO_2_) was assessed polarographically using a flexible O_2_-sensitive catheter electrode (length of the O_2_-sensitive cathode 5 mm, outer catheter diameter 0.35 mm, LICOX, GMS, Kiel-Mielkendorf, Germany) which was inserted into the centre of the tumour for continuous monitoring of tumour pO_2_. Before each experiment, the pO_2_ electrode was calibrated with room air in a chamber with constant temperature, taking the ambient barometric pressure into account.

After the surgical procedure, animals were allowed to stabilise and measurements commenced once constant baseline readings for PP, RBC flux and tumour oxygen tension were obtained for at least 20 min (if constant baseline readings could not be achieved values were excluded from further data analysis). Thereafter baseline values for blood flow rate, PP, RBC flux and tumour pO_2_ were continuously recorded before the commencement of nifedipine or vehicle infusion and throughout the 30 min infusion period. Arterial and venous perfusate samples were taken at t=0 (immediately prior to ILP), 15, and 30 min to assess pH/blood gas status (type ABL 5, Radiometer, Copenhagen, Denmark) as well as glucose and lactate concentrations which were determined enzymatically using standard test kits (1442457 and 256773; Boehringer-Mannheim, Mannheim, Germany).

### Metabolite concentrations

In an additional series of experiments, the tumour of the isolated perfused leg and of the contralateral hind limbs of the anaesthetised animals were surgically removed and rapidly frozen in liquid nitrogen immediately following termination of the perfusion procedure (30 min of equilibration plus 30 min of nifedipine or vehicle infusion) and the tumours subsequently removed. The tumours were ground to a fine powder and subsequently freeze-dried. Thereafter, glucose and lactate concentrations were assayed enzymatically using standard test kits (1442457 and 256773; Boehringer-Mannheim, Mannheim, Germany). Concentrations of adenosine triphosphate (ATP), adenosine diphosphate (ADP) and adenosine monophosphate (AMP) were determined by high-performance liquid chromatography (HPLC, for details see Krüger *et al*, 1991). In brief, 2–3 mg aliquots of freeze-dried tissue were extracted with 0.3 M perchloric acid, centrifuged and the supernatant neutralised with 2 M potassium hydroxide and diluted 1 : 2 with the mobile HPLC-phase. Concentrations were then determined using reversed-phase high-performance liquid chromatography (HPLC) and UV-detection at 254 nm. The isocratic separation was performed by a Superspher RP 18 end-capped column (250×4 mm; Knauer, Berlin Germany) and a guard cartridge system (5×4 mm). The mobile phase consisted of 0.05 M ammonium dihydrogen phospate, 0.01 M tetrabutylammonium hydroxide and 11.5% acetonitrile (v v^−1^), adjusted to pH 6.4. The flow rate was 0.9 ml min^−1^ and the sample size 40 μl. Concentrations of all metabolites are expressed as μmol g^−1^ tissue wet weight. Wet weight was estimated from tissue samples as follows: tumours (perfused for 1 h) were excised from the hind foot dorsum, skin was removed and wet weight recorded. Tumours were dried at 60°C until constant weight readings were attained. The tissue water content was the same in perfused and in untreated tumours (82.1±0.2% *vs* 82.0±0.1%) of comparable volume. Oedema formation during ILP can thus be excluded.

### Statistical analysis

Results are expressed as means±s.e.m. unless stated otherwise. Differences between groups were assessed by the two-tailed Wilcoxon test for paired or unpaired samples as appropriate. The significance level was set at α=5% for all comparisons.

## RESULTS

**Figure 2:**
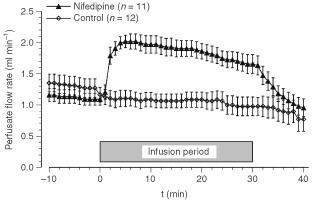
Perfusate flow rate during isolated limb perfusion upon application of nifedipine or vehicle alone (gray bar). Data represent mean±s.e.m. (*n*=number of perfusion experiments).

Baseline PP in the isolated-perfused leg before administration of nifedipine or vehicle was 141±8 and 131±7 mmHg, respectively, at comparable baseline perfusate flow rates of 1.09±0.10 ml min^−1^ in the nifedipine group and of 1.16±0.12 ml min^−1^ in control animals. Upon nifedipine application, PP initially dropped (with lowest values at t=3 min) to 81% of the baseline value. Thereafter however, the decrease was intentionally compensated by the increased perfusate flow rate resulting in a constant PP of between 130 and 135 mmHg. During nifedipine application, the *perfusate flow rate* had to be increased by almost 100% in order to maintain a constant PP (Figure 2

), suggesting a pronounced nifedipine-induced vasodilation either in the tumour or in the normal tissue of the perfused hind limb (e.g., skeletal muscle). After discontinuation of nifedipine infusion, the PP increased within 5 min so that the perfusate rate had to be reduced to achieve pressure-constant conditions (Figure 2). Application of the vehicle alone resulted in only a slight decrease in perfusion pressure by 2% of the baseline value so that the perfusate flow rate remained almost constant during vehicle administration (Figure 2).

**Figure 3:**
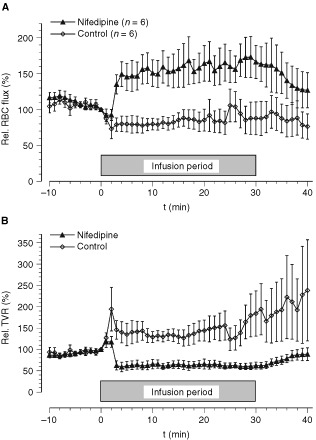
(**A**) RBC-flux and (**B**) resistance to flow (TVR) during isolated limb perfusion upon application of nifedipine. Data represent mean±s.e.m. (*n*=number of tumours investigated; the RBC flux value of each tumour was obtained by calculating the mean values from up to three individual probes located in central and peripheral regions of the tumour).

At constant PP during nifedipine application, the mean *RBC flux in the tumours* increased by approximately 60% whereas the TVR was reduced by 40% indicating a vasodilation of vessels feeding the tumour (Figure 3

). In control animals, the opposite was observed, with a moderate reduction in RBC flux and an increase in vascular resistance revealing a slight vasoconstriction upon vehicle application.

**Figure 4:**
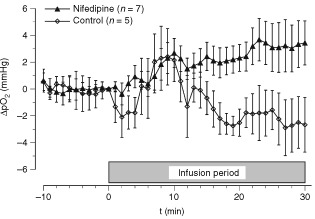
Changes of mean tumour pO_2_ during isolated limb perfusion upon application of nifedipine compared to the pO_2_ value immediately prior to the commencement of drug infusion. Data represent mean±s.e.m. (*n*=number of tumours investigated).

Since the oxygen content of the arterial perfusate was maintained by oxygenising the blood with a capillary oxygenator to give an oxyhaemoglobin saturation of almost 100%, the increase in perfusate flow rate during nifedipine application resulted in a pronounced *increase in O_2_ delivery* to the perfused limb. However, the improved supply had only a minor impact on the O_2_ partial pressure of the tumour tissue. Figure 4

illustrates that during nifedipine infusion only a minor increase in the mean tumour pO_2_ of approximately 2 mmHg occurred. The application of the vehicle alone resulted in a slight worsening in mean tumour pO_2_ (a decrease of up to 3 mmHg, Figure 4) but taking the pronounced inter-tumour variability of the oxygenation changes into account, these differences were not statistically significant. The improved O_2_ supply therefore did not result in an improvement of tumour oxygenation. Since the oxygenation status of a tissue results from a dynamic steady state between O_2_ supply and O_2_ uptake, one possible explanation of this result might be an increased O_2_ utilisation during nifedipine infusion. Although the O_2_ supply was nearly doubled by nifedipine application, the arterio-venous O_2_ concentration difference (avDO_2_) remained almost constant (Table 1

**Table 1:**
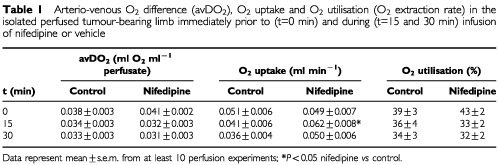
Arterio-venous O_2_ difference (avDO_2_), O_2_ uptake and O_2_ utilisation (O_2_ extraction rate) in the isolated perfused tumour-bearing limb immediately prior to (t=0 min) and during (t=15 and 30 min) infusion of nifedipine or vehicle

), indicating a significant increase in O_2_ uptake following the improved O_2_ delivery caused by nifedipine administration. If all experiments (nifedipine treatment and controls) were taken together, a linear correlation (r^2^=0.606) was seen between the O_2_ supply to the tumour-bearing leg and the O_2_ uptake into the tumour (Figure 5

**Figure 5:**
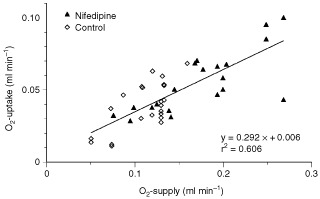
Correlation between O_2_ supply and O_2_ uptake during isolated limb perfusion.

), a phenomenon which might explain the lack of a substantial tumour pO_2_ increase during nifedipine administration.

As a result of the reduced haematocrit (25%) used in the perfusate, the oxygen supply during ILP *per se* seems to be somewhat restricted resulting in a higher glycolytic rate as indicated by a lower glucose concentration in ILP tumours without nifedipine application (0.59±0.09 compared to 1.11±0.22 μmol g^−1^ in the contralateral control tumours which were not isolated-perfused), and higher lactate levels in the isolated perfused limb tumours (27.0±3.5 *vs* 9.3±1.7 μmol g^−1^ in the contralateral leg). However, the higher glycolytic rate during ILP did not have a strong impact on the bioenergetic status. In isolated perfused tumours (without nifedipine) the ATP levels were 0.66±0.08 compared to 1.18±0.13 μmol g^−1^ in the contralateral non-isolated-perfused tumours. Although the oxygen supply was restricted during ILP *per se* (due to the reduced haematocrit of the perfusate which results in a lower oxygen transport capacity), a stable bioenergetic status was maintained.

With nifedipine infusion during ILP, tumour perfusion substantially increased and resulted in a considerably higher nutrient supply which was reflected by a higher (though not statistically significant) tumour glucose concentration (1.39±0.39 μmol g^−1^ during nifedipine application *vs* 0.59±0.09 μmol g^−1^ in ILP tumours without nifedipine). However, since the oxygenation status was not improved by nifedipine neither the lactate levels (25.7±2.2 *vs* 27.0±3.5 μmol g^−1^) nor the ATP concentration (0.79±0.11 *vs* 0.66±0.08 μmol g^−1^) markedly changed during nifedipine treatment compared to tumours during ILP without nifedipine. Obviously, the increase in tumour blood flow had practically no impact on the metabolic or bioenergetic status of the tumour.

## DISCUSSION

Pressure-controlled ILP allows the administration of anti-cancer agents to a tumour at high doses with reduced systemic toxicity. However, due to the compromised microcirculation found in many experimental and human tumours a sub-optimum delivery (pharmacokinetics) of chemotherapeutic agents can be expected. In addition, the deterioration and heterogeneity of tumour blood flow is responsible for hypoxia in tumours which in turn reduces the efficacy of oxygen-dependent chemotherapeutic agents. For this reason, a supportive treatment modality (e.g., nifedipine application) which leads to a reduction of heterogeneity or an improvement of tumour blood flow and/or a reduction of hypoxia might be of clinical interest.

### Tumour perfusion

The effect of CCBs on tumour perfusion has been investigated extensively over the last two decades. Wood and Hirst (1989) described dose-dependent effects of different types of CCBs on tumour perfusion and radiosensitivity. While verapamil, nifedipine and diltiazem enhanced radiosensitivity at low doses and increased radioresistance at higher doses, flunarizine, which exhibits only limited suppression of cardiac contractility (Robertson and Robertson, 1996), increased radiosensitivity at all dose levels. Pressure-controlled isolated perfusion permits the use of all classes of CCBs irrespective of their suppression of cardiac contractility since PP can be maintained by adjusting the perfusate flow rate. Vessels feeding the tumour dilate upon application of CCBs, while microvessels within hypoxic tumours might not be able to react adequately to vasodilatory stimuli since in their acidic and hypoxic microenvironment they are already maximally dilated (Vaupel *et al*, 1989) or are lacking a functional smooth muscle layer. Thus an increased flow rate will result in an enhanced perfusion in both normal and tumour tissue as long as the vascular beds of these tissues lie in series with one another. If both vessels are located parallel to each other, dilation of the host tissue vessels (and not of the tumour vasculature) may induce a redirection of blood flow in favour of the surrounding normal tissue (‘steal’ phenomenon). However, the results of the present study show that the nifedipine-induced increase in perfusion rate actually leads to an increased tumour blood flow. Perfusate flow rate and tumour RBC flux (as measured by the laser Doppler technique) increased almost in parallel, except towards the end of the infusion period when the perfusate flow was reduced by approximately 30% (Figure 2), whereas the RBC flux showed a sustained increased value (Figure 3A) indicating a vasodilatatory effect of nifedipine during this period (Figure 3B). Tumour perfusion increased during nifedipine application by 50–70% of the pre-treatment value. These data clearly indicate that nifedipine-induced vasodilation does not induce a ‘steal’ effect of the surrounding normal tissue of the isolated perfused leg (e.g., skeletal muscle). The improvement of tumour blood flow seen in the present experiments was comparable to that found in other studies where increases ranged from approximately +30% to a maximum of +200% using various kinds of CCBs (Kaelin *et al*, 1984; Vaupel and Menke, 1987; Wood and Hirst, 1989; Dewhirst *et al*, 1992; Zenke *et al*, 1996; Muruganandham *et al*, 1999). Wu *et al* (1997) demonstrated that an enhanced flow rate is accompanied by improved pharmacokinetics of the anti-cancer drug melphalan. Raising the perfusate flow rate from 4 to 8 ml min^−1^ led to a two-fold increase in melphalan concentration in the tumour tissue. Zenke *et al* (1996) showed that co-administration of diltiazem and the anti-cancer drug nimustine resulted in a 39% increase in intra-tumoural blood flow and a higher concentration of nimustine in rat gliomas. The improved microcirculation seen upon application of nifedipine as shown in our study may therefore present a useful and appropriate means of promoting the delivery of anti-cancer agents to the tumour tissue.

### Tumour oxygenation and oxygen utilisation

In the present study, pO_2_ values measured in tumours of isolated perfused limbs were lower (median pO_2_: 2 mmHg, fraction of hypoxic pO_2_ values ⩽2.5 mmHg: 62%) than in non-perfused contralateral control tumours with comparable volumes (median pO_2_: 4–13 mmHg, fraction of hypoxic pO_2_ values: 10–40%) (Kelleher and Vaupel, 1993; Kelleher *et al*, 1995, 1998a). These data indicate that during isolated limb perfusion O_2_-supply conditions were restricted (compared to control conditions in non-perfused limbs). This is partially due to the lower cHb of the perfusate (74±2 g l^−1^) compared to whole blood. Control measurements in the normal subcutis of perfused hind limbs (non-tumour bearing) and non-artificially perfused legs also showed poorer oxygenation during ILP (median pO_2_=37 mmHg in subcutis during ILP and 49 mmHg in control legs). In many experimental ILP studies (Bonen *et al*, 1994) as well as in the clinical setting, perfusates with reduced cHb (compared to normal whole blood) or even erythrocyte-free perfusates were used. Enhanced flow rates (e.g., during nifedipine application) might therefore (at least partially) compensate for the reduced oxygen transport capacity associated with a low cHb.

Many studies have demonstrated an increased tumour blood flow upon application of CCBs but the postulated effect on tumour oxygenation has generally not been documented. Based on mathematical simulation experiments, Secomb *et al* (1995) proposed that a fraction of hypoxic pO_2_-values of 30% would only be abolished if the flow rate were to be increased by a factor of 4 or more. An improvement in tumour oxygenation was reported in two studies by Dewhirst *et al* (1992) and Muruganandham *et al* (1999) where tumour blood flow increased to two- and three-fold the baseline values, respectively. Using a dorsal skin flap preparation Dewhirst *et al* (1992) demonstrated that flunarizine enhanced perivascular pO_2_ by approximately 50% in the tumour center (pO_2_ prior to drug administration 25 mmHg, with an increase of 12 mmHg). Muruganandham *et al* (1999) reported a diltiazem-induced improvement in tumour oxygenation of 25% in a subcutaneously growing tumour. The present study failed to show a marked effect of nifedipine on tumour oxygenation during ILP. Although tumour perfusion increased by 50% during nifedipine application (Figure 3A), the mean tumour pO_2_ rose only slightly by 2–3 mmHg (Figure 4). These results are still in accordance with previous studies demonstrating that CCBs can affect the oxygen consumption rate of tumour cells (Biaglow *et al*, 1986; Vaupel and Mueller-Klieser, 1986). These studies showed that with high concentrations of verapamil and other CCBs the O_2_ utilisation in several tumour cell lines can be reduced under *in vitro* conditions by up to 30%. However, *in vivo*, the tissue concentrations of these drugs are presumably much lower inducing a less pronounced reduction. In addition, previous studies also clearly demonstrated that a reduction of O_2_ consumption rate by only 30% is not sufficient to cause a significant improvement in the oxygenation status of a tumour (Thews *et al*, 1999). For this reason, the results of the present study do not necessarily contradict previously published *in vitro* data.

Since the oxygenation status of a tissue results from a dynamic steady-state between the oxygen supply and the cellular O_2_ consumption, one possible explanation for the lack of oxygenation improvement during nifedipine application may be an increase in O_2_ uptake during the treatment. Calculating the oxygen uptake from the avDO_2_ and the perfusion rate, it became obvious that with a greater oxygen supply (as a result of a higher perfusion rate during nifedipine application) the O_2_-uptake by the tumour increased linearly (Figure 5). Previous studies by Gullino *et al* (1967a,b) and Kallinowski *et al* (1989a,b) showed that the tumour O_2_ consumption is dependent on the oxygen availability as long as a ‘saturation level’ of the O_2_ supply is not reached. An improvement in the convective O_2_ transport (by increased perfusion) will only reduce tumour hypoxia if the O_2_ supply greatly exceeds the consumption rate. Due to the reduced haemoglobin level during ILP, the oxygen transport capacity of the perfusate is diminished (0.11 ml O_2_ per ml blood during ILP *vs* 0.2 ml O_2_ per ml blood in controls) resulting in a restricted supply situation. Obviously, the O_2_ supply in the present study was markedly lower than the ‘saturation level’ and the convective O_2_ transport during ILP did not meet the demands of the tumour tissue. During ILP therefore, where the O_2_ transport capacity is reduced, an improvement in the perfusion rate of 50–100% is probably not sufficient to bring about a significant increase in the median tumour pO_2_.

As a result of the restricted oxygen supply during ILP (due to the reduced haematocrit of the perfusate), the lactate levels in tumours of isolated perfused limbs were significantly higher than in tumours of contralateral control limbs, indicating a much higher glycolytic rate. However, the higher glycolytic rate had only a minor impact on the bioenergetic status resulting in ATP levels which are comparable to those found previously in untreated tumours (Vaupel *et al*, 1994). Although nifedipine application during ILP increased tumour perfusion by approximately 60% (Figure 3) and in turn the nutrient and oxygen supply, only the glucose concentration in the tumour was elevated. Nifedipine had almost no impact on the bioenergetic status, a finding which is in good accordance to an earlier study demonstrating the energy status to be relatively stable despite substantial changes in blood flow and tissue oxygenation providing tumour perfusion does not fall below a certain threshold (Vaupel *et al*, 1994).

In conclusion, nifedipine can significantly improve tumour perfusion during pressure-controlled ILP. ‘Steal phenomena’ in favour of the surrounding normal tissue and oedema formation were not observed. Nifedipine can enhance tumour microcirculation and may therefore promote the delivery (pharmacokinetics) of anti-cancer agents. Although the application of this calcium channel blocker increases oxygen availability to the tumour the improvement of perfusion by nifedipine does not result in a substantial reduction of tumour hypoxia. On the basis of these results, nifedipine application during ILP can be expected to increase the delivery of anti-cancer drugs to the tumour and by this improve the efficacy of pressure-controlled ILP.

## References

[bib1] BiaglowJEVarnesMEJacobsonBSuitHD1986 Effect of calcium channel blocking drugs on tumor cell oxygen utilization Adv Exp Med Biol 200 583589 3799350 10.1007/978-1-4684-5188-7_71

[bib2] BonenAClarkMGHenriksenEJ1994 Experimental approaches in muscle metabolism: hindlimb perfusion and isolated muscle incubations Am J Physiol 266 E1E16 8304435 10.1152/ajpendo.1994.266.1.E1

[bib3] CornwellMMPastanIGottesmanMM1987 Certain calcium channel blockers bind specifically to multidrug-resistant human KB carcinoma membrane vesicles and inhibit drug binding to P-glycoprotein J Biol Chem 262 21662170 2434476

[bib4] CreechOKrementzETRyanRFWinbladJN1958 Chemotherapy of cancers: regional perfusion utilizing an extracorporeal circuit Ann Surg 148 616631 13583933 10.1097/00000658-195810000-00009 PMC1450870

[bib5] de WiltJHManusamaERvan TielSTvan IjkenMGten HagenTLEggermontAM1999 Prerequisites for effective isolated limb perfusion using tumour necrosis factor alpha and melphalan in rats Br J Cancer 80 161166 10389992 10.1038/sj.bjc.6690335 PMC2362986

[bib6] DewhirstMWOngETMadwedDKlitzmanBSecombTBrizelDBonaventuraJRosnerGKavanaghBEdwardsJ1992 Effects of the calcium channel blocker flunarizine on the hemodynamics and oxygenation of tumor microvasculature Radiat Res 132 6168 1410275

[bib7] EggermontAMSchraffordt-KoopsHLienardDKroonBBvan GeelANHoekstraHJLejeuneFJ1996 Isolated limb perfusion with high-dose tumor necrosis factor-alpha in combination with interferon-gamma and melphalan for nonresectable extremity soft tissue sarcomas: a multicenter trial J Clin Oncol 14 26532665 8874324 10.1200/JCO.1996.14.10.2653

[bib8] FontijneWPMookPHElstrodtJMSchraffordt-KoopsHOldhoffJWildevuurCR1985a Isolated hindlimb perfusion in dogs: the effect of perfusion pressures on the oxygen supply (ptO_2_ histogram) to the skeletal muscle Surgery 97 278284 3975848

[bib9] FontijneWPMookPHKoopsHSOldhoffJWildevuurCR1985b Improved tissue perfusion during pressure regulated hyperthermic regional isolated perfusion. A clinical study Cancer 55 14551461 3978538 10.1002/1097-0142(19850401)55:7<1455::aid-cncr2820550706>3.0.co;2-1

[bib10] GullinoPMGranthamFHCourtneyAH1967a Utilization of oxygen by transplanted tumors *in vivo* Cancer Res 27 10201030 4290856

[bib11] GullinoPMGranthamFHCourtneyAHLosonczyI1967b Relationship between oxygen and glucose consumption by transplanted tumors *in vivo* Cancer Res 27 10411052 4290858

[bib12] HelsonL1984 Calcium channel blocker enhancement of anticancer drug cytotoxicity – a review Cancer Drug Deliv 1 353361 6100477 10.1089/cdd.1984.1.353

[bib13] HillRPStirlingD1987 Oxygen delivery and tumour responseInRadiation Research, Proceedings of the 8th International Congress of Radiation ResearchFielden EM, Fowler JF, Hendry JH, Scott D (eds)pp725730London: Taylor & Francis

[bib14] HohenbergerPKettelhackC1998 Clinical management and current research in isolated limb perfusion for sarcoma and melanoma Oncology 55 89102 9499194 10.1159/000011842

[bib15] HonnKVOnodaJMPampalonaKBattagliaMNeagosGTaylorJDDiglioCASloaneBF1985 Inhibition by dihydropyridine class calcium channel blockers of tumor cell-platelet-endothelial cell interactions in vitro and metastasis in vivo Biochem Pharmacol 34 235241 3966924 10.1016/0006-2952(85)90130-3

[bib16] HöckelMVaupelP2001 Tumor hypoxia: definitions and current clinical, biologic, and molecular aspects J Natl Cancer Inst 93 266276 11181773 10.1093/jnci/93.4.266

[bib17] JirtleRL1988 Chemical modification of tumour blood flow Int J Hyperthermia 4 355371 3290350 10.3109/02656738809016490

[bib18] KaelinWGShrivastavSJirtleRL1984 Blood flow to primary tumors and lymph node metastases in SMT-2A tumor-bearing rats following intravenous flunarizine Cancer Res 44 896899 6692410

[bib19] KallinowskiFSchlengerKHKloesMStohrerMVaupelP1989a Tumor blood flow: the principal modulator of oxidative and glycolytic metabolism, and of the metabolic micromilieu of human tumor xenografts in vivo Int J Cancer 44 266272 2759732 10.1002/ijc.2910440214

[bib20] KallinowskiFSchlengerKHRunkelSKloesMStohrerMOkunieffPVaupelP1989b Blood flow, metabolism, cellular microenvironment, and growth rate of human tumor xenografts Cancer Res 49 37593764 2736517

[bib21] KelleherDKEngelTVaupelPW1995 Changes in microregional perfusion, oxygenation, ATP and lactate distribution in subcutaneous rat tumours upon water-filtered IR-A hyperthermia Int J Hyperthermia 11 241255 7790738 10.3109/02656739509022460

[bib22] KelleherDKNauthCThewsOKruegerWVaupelP1998a Localized hypothermia: Impact on oxygenation, microregional perfusion, metabolic and bioenergetic status of subcutaneous rat tumours Br J Cancer 78 5661 9662251 10.1038/bjc.1998.442 PMC2062945

[bib23] KelleherDKThewsOVaupelP1998b Regional perfusion and oxygenation of tumors upon methylxanthine derivative administration Int J Radiat Oncol Biol Phys 42 861864 9845111 10.1016/s0360-3016(98)00318-6

[bib24] KelleherDKVaupelP1993 Nicotinamide exerts different acute effects on microcirculatory function and tissue oxygenation in rat tumors Int J Radiat Oncol Biol Phys 26 95102 8482636 10.1016/0360-3016(93)90178-x

[bib25] KrügerWMayerWKSchaeferCStohrerMVaupelP1991 Acute changes of systemic parameters in tumour-bearing rats, and of tumour glucose, lactate, and ATP levels upon hyperthermia and/or hyperglycaemia J Cancer Res Clin Oncol 117 409415 1909697 10.1007/BF01612759 PMC12201855

[bib26] MuruganandhamMKasiviswanathanAJagannathanNRRaghunathanPJainPCJainV1999 Diltiazem enhances tumor blood flow: MRI study in a murine tumor Int J Radiat Oncol Biol Phys 43 413421 10030270 10.1016/s0360-3016(98)00403-9

[bib27] NagelKGhussenFKrügerIIsselhardW1987 Miniature equipment for the perfusion of rat limbs Res Exp Med 187 18 10.1007/BF01854962 3575879

[bib28] OmlorGHVaupelPAlexanderC(eds)1995 Isolated hyperthermic limb perfusion.Berlin: Springer

[bib29] OnodaJMNelsonKKTaylorJDHonnKV1989 In vivo characterization of combination antitumor chemotherapy with calcium channel blockers and cis-diamminedichloroplatinum(II) Cancer Res 49 28442850 2720644

[bib30] PistersPWFeigBWLeungDHBrennanMF1997 New developments in soft tissue sarcoma Cancer Treat Res 90 91107 9367079 10.1007/978-1-4615-6165-1_5

[bib31] RobertsonRMRobertsonD1996 Drugs used for the treatment of myocardial ischemiaInGoodman & Gilman's The pharmacological basis of therapeuticsHardman JG, Goodman Gilman A, Limbird LE (eds)pp759779New York: McGraw-Hill

[bib32] Schraffordt-KoopsHEggermontAMLienardDKroonBBHoekstraHJvan GeelANNiewegOELejeuneFJ1998 Hyperthermic isolated limb perfusion with tumour necrosis factor and melphalan as treatment of locally advanced or recurrent soft tissue sarcomas of the extremities Radiother Oncol 48 14 9756165 10.1016/s0167-8140(98)00040-1

[bib33] SecombTWHsuROngETGrossJFDewhirstMW1995 Analysis of the effects of oxygen supply and demand on hypoxic fraction in tumors Acta Oncol 34 313316 7779415 10.3109/02841869509093981

[bib34] SeynhaeveALBde WiltJHWvan TielSTEggermontAMMten HagenTLM2002 Isolated limb perfusion with actinomycin D and TNF-alpha results in improved tumour response in soft-tissue sarcoma-bearing rats but is accompanied by severe local toxicity Br J Cancer 86 11741179 11953868 10.1038/sj.bjc.6600169 PMC2364186

[bib35] SmitsGJRomanRJLombardJH1986 Evaluation of laser-Doppler flowmetry as a measure of tissue blood flow J Appl Physiol 61 666672 2943717 10.1152/jappl.1986.61.2.666

[bib36] ThewsOKelleherDKHummelMVaupelP1999 Can tumor oxygenation be improved by reducing cellular oxygen consumption? Adv Exp Med Biol 471 525532 10659186 10.1007/978-1-4615-4717-4_62

[bib37] VaupelPKallinowskiFOkunieffP1989 Blood flow, oxygen and nutrient supply, and metabolic microenvironment of human tumors: a review Cancer Res 49 64496465 2684393

[bib38] VaupelPKelleherDKEngelT1994 Stable bioenergetic status despite substantial changes in blood flow and tissue oxygenation in a rat tumour Br J Cancer 69 4649 8286209 10.1038/bjc.1994.7 PMC1968765

[bib39] VaupelPMenkeH1987 Blood flow, vascular resistance and oxygen availability in malignant tumours upon intravenous flunarizine Adv Exp Med Biol 215 393398 3673743 10.1007/978-1-4684-7433-6_48

[bib40] VaupelPMenkeH1989 Effect of various calcium antagonists on blood flow and red blood cell flux in malignant tumors Progr Appl Microcirc 14 88103

[bib41] VaupelPMueller-KlieserW1986 Verapamil inhibits the respiration rate of cancer cells Adv Exp Med Biol 200 645648 3799356 10.1007/978-1-4684-5188-7_77

[bib42] VaupelPThewsOHoeckelM2001 Treatment resistance of solid tumors – Role of hypoxia and anemia Med Oncol 18 243259 11918451 10.1385/MO:18:4:243

[bib43] WoodPJHirstDG1988 Cinnarizine and flunarizine as radiation sensitisers in two murine tumours Br J Cancer 58 742745 3224079 10.1038/bjc.1988.301 PMC2246854

[bib44] WoodPJHirstDG1989 Modification of tumour response by calcium antagonists in the SCVII/St tumour implanted at two different sites Int J Radiat Biol 56 355367 2570820 10.1080/09553008914551511

[bib45] WorkmanPTwentymanPBalkwillFBalmainAChaplinDJDoubleJAEmbletonJNewellDRaymondRStablesJStephensTWallaceJ1998 United Kingdom Co-ordinating Committee on Cancer Research (UKCCCR) Guidelines for the Welfare of Animals in Experimental Neoplasia (Second Edition) Br J Cancer 77 110 10.1038/bjc.1998.1 PMC2151254 9459138

[bib46] WuZYSmithersBMParsonsPGRobertsMS1997 The effects of perfusion conditions on melphalan distribution in the isolated perfused rat hindlimb bearing a human melanoma xenograft Br J Cancer 75 11601166 9099965 10.1038/bjc.1997.200 PMC2222787

[bib47] ZenkeKNakagawaKKumonYOhtaSHatakeyamaTSakakiS1996 A strategy for selective anti-cancer drug concentration increase in rat glioma tissue with Ca^2+^-channel blocker co-administration: calcium kinetics in intra-glioma arteriolar smooth muscle cells J Neurooncol 30 2536 8865000 10.1007/BF00177440

